# RNA-Binding Proteins Driving the Regulatory Activity of Small Non-coding RNAs in Bacteria

**DOI:** 10.3389/fmolb.2020.00078

**Published:** 2020-05-13

**Authors:** Ana P. Quendera, André F. Seixas, Ricardo F. dos Santos, Inês Santos, João P. N. Silva, Cecília M. Arraiano, José M. Andrade

**Affiliations:** Instituto de Tecnologia Química e Biológica António Xavier, Universidade Nova de Lisboa, Oeiras, Portugal

**Keywords:** RNA-binding proteins, RNA chaperone, ribonucleases, small non-coding RNAs, CsrA, Hfq, ProQ

## Abstract

Small non-coding RNAs (sRNAs) are critical post-transcriptional regulators of gene expression. Distinct RNA-binding proteins (RBPs) influence the processing, stability and activity of bacterial small RNAs. The vast majority of bacterial sRNAs interact with mRNA targets, affecting mRNA stability and/or its translation rate. The assistance of RNA-binding proteins facilitates and brings accuracy to sRNA-mRNA basepairing and the RNA chaperones Hfq and ProQ are now recognized as the most prominent RNA matchmakers in bacteria. These RBPs exhibit distinct high affinity RNA-binding surfaces, promoting RNA strand interaction between a *trans*-encoding sRNA and its mRNA target. Nevertheless, some organisms lack ProQ and/or Hfq homologs, suggesting the existence of other RBPs involved in sRNA function. Along this line of thought, the global regulator CsrA was recently shown to facilitate the access of an sRNA to its target mRNA and may represent an additional factor involved in sRNA function. Ribonucleases (RNases) can be considered a class of RNA-binding proteins with nucleolytic activity that are responsible for RNA maturation and/or degradation. Presently RNase E, RNase III, and PNPase appear to be the main players not only in sRNA turnover but also in sRNA processing. Here we review the current knowledge on the most important bacterial RNA-binding proteins affecting sRNA activity and sRNA-mediated networks.

## Introduction

The majority of small non-coding RNAs (sRNAs) interact with a complementary mRNA through an antisense mechanism, leading to the formation of a duplex sRNA-mRNA region. Consequently, expression from the target mRNA is affected and frequently repressed (Storz et al., 2011). sRNA-mediated networks are cost efficient and often more rapid in the reprogramming of gene expression than pathways that rely exclusively on regulatory proteins (Shimoni et al., 2007). However, the interaction between sRNAs and RNA-binding proteins (RBPs) is often critical for the regulatory activity of sRNAs. RNA-binding proteins are a diverse class of proteins ubiquitously found in all living organisms and that control all steps of the life of an RNA (Smirnov et al., 2017a). The capacity of these proteins to recognize and bind RNA molecules arises from the presence of well-defined RNA-binding domains, such as the canonical S1 domain, cold shock domain (CSD), K homology (KH) domain, amongst others (Holmqvist and Vogel, 2018). Additional regions may also contribute to RNA-protein interactions, like the disordered regions that confer flexibility to proteins. The overall fold of the protein and the recognition of different RNA-binding motifs determines the interaction with RNA in a sequence- and/or structure-specific dependent manner. RBPs and sRNAs networks have been extensively studied in Eukarya and Bacteria, with a current lack of information about this regulation in Archaea (Gelsinger and DiRuggiero, 2018). Though many RBPs can be found in bacteria only few have been shown to associate with sRNAs. However, these participate in a variety of reactions that affect the catalytic and molecular recognition properties of sRNAs.

**FIGURE 1:**
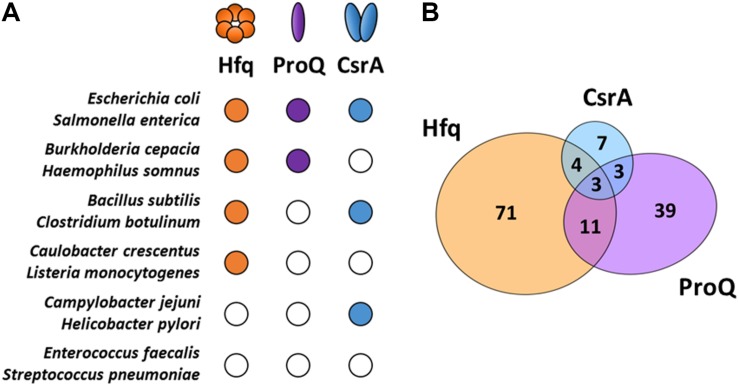
Major RNA chaperones in bacteria. **(A)** RNA chaperone distribution among representative bacteria. The *Escherichia coli* RNA chaperones Hfq, ProQ, and CsrA sequences were used as reference for comparison with other species using the co-occurrence analysis of the STRING database (Szklarczyk et al., 2017). Representative organisms were selected to illustrate the multiple combinations of RNA chaperones expression among bacteria. Close/open circles indicate presence/absence, respectively. **(B)** Venn diagram showing the intersection of sRNA substrates for each one of the RNA chaperones (Hfq, ProQ, and CsrA) in *Salmonella enterica*. Information retrieved from CLIP-seq datasets from Holmqvist et al. (2016, 2018).

RNA chaperones constitute a specific group of RBPs that transiently bind and induce structural changes in RNA substrates by melting RNA secondary structures (Woodson et al., 2018). Such structural rearrangements influence not only the stability of sRNA and mRNA molecules but also facilitate the basepairing of sRNAs and mRNAs. Moreover, RNA chaperones that bind simultaneously the sRNA and the target mRNA, bring them closely together in a complex, promoting the annealing and formation of stable RNA-RNA interactions. Though sRNA-mRNA basepairing can occur in the absence of RNA chaperones, their presence greatly accelerates this process (Rajkowitsch and Schroeder, 2007; Panja et al., 2013). Three major RNA chaperones that assist sRNA function in bacteria are currently known: the Sm family member Hfq (Santiago-Frangos and Woodson, 2018), the FinO family member ProQ (Smirnov et al., 2016) and the prototype of its family CsrA (Müller et al., 2019). Despite being widespread, these RBPs are not evenly present in bacteria and the interactome studies of these RNA chaperones indicate they preferably bind different sRNAs (Figure 1), suggesting more specialized roles for each of them (Holmqvist et al., 2016, 2018; Smirnov et al., 2016; Melamed et al., 2019).

Ribonucleases (RNases) are another group of specific RBPs that interact with sRNAs. These enzymes are responsible for the catalytic cleavage of all classes of RNA (Arraiano et al., 2010). The stability of sRNAs results from the interplay between RNA chaperones and RNases with the sRNAs, as RNA chaperones may protect or expose the sRNAs to the nucleolytic action of RNases (Holmqvist and Vogel, 2018). The main RNases implicated in sRNA turnover are the endonucleases RNase E and RNase III and the exonuclease PNPase (Saramago et al., 2014). In this mini review, we summarize the current information on the major RNA chaperones and RNases governing the activity of bacterial sRNAs.

## RNA Chaperones

### Hfq

Hfq is widely recognized as a global regulator and key element of sRNA-based networks. Hundreds of sRNA molecules have been reported in *Escherichia coli*, and ∼30% rely on Hfq to carry on their functions (Vogel and Luisi, 2011). Hfq is particularly important for the action of *trans*-encoded sRNAs (which are expressed from a different genomic region than their mRNA targets), stabilizing the imperfect basepairing between sRNA/mRNA pairs. At least in Gram-negative bacteria, Hfq primary role is to promote the annealing of sRNA-mRNA duplexes, acting as a molecular “matchmaker,” but its role in Gram-positive bacteria is more controversial (Woodson et al., 2018; Dos Santos et al., 2019). Interestingly, Hfq can bind other substrates including rRNA (Andrade et al., 2018), tRNA (Lee and Feig, 2008), and even DNA molecules (Cech et al., 2016). The wide substrate selection of Hfq suggests additional functions for this protein in the cell, such as its involvement in ribosome biogenesis and translation fidelity (Andrade et al., 2018), and DNA compaction (Jiang et al., 2015; Malabirade et al., 2017). Hfq is also involved in many protein-protein interactions (Caillet et al., 2019), namely with proteins involved in RNA degradation, such as RNase E (Morita et al., 2005), PNPase (Mohanty et al., 2004), and Poly(A) Polymerase (Le Derout et al., 2003). These Hfq-based complexes hint a close relationship between Hfq and the RNA degradation machinery.

Interactome studies identified thousands of Hfq-bound RNA pairs, dominated by mRNA-sRNA pairs exhibiting sequence complementarity (Holmqvist et al., 2016; Melamed et al., 2016; Mihailovic et al., 2018). Hfq binds single-stranded RNA showing a preference for (ARN)_*n*_ motifs frequently found on mRNA (Link et al., 2009) and short poly(U) tails present at the 3′-end of sRNAs (Sauer and Weichenrieder, 2011; Sauer et al., 2012). Hfq binds transiently to sRNA and mRNA, dissociating from the RNA duplex soon after basepairing occurs (Fender et al., 2010; Hwang et al., 2011). This widely conserved protein is composed by two N-terminal structural motifs (Sm1 and Sm2) and a variable intrinsically disordered C-terminal tail. The Hfq Sm motifs are characteristic of the Sm/Lsm family of RNA-binding proteins (Møller et al., 2002). Members of this family typically adopt a multimer ring-like architecture, with Hfq assembling into an homohexamer. Multiple RNA-binding surfaces are consequently present in the Hfq ring: the proximal face, the distal face, the lateral rim and the C-terminal tail. The RNA is wrapped around the ring, causing reshaping of the RNA’s secondary structure and/or bringing two different strands of RNA into close proximity (Woodson et al., 2018).

sRNAs have been classified in two major classes according to their dependence on Hfq contact surfaces. The vast majority of bacterial sRNAs belong to class I. Hfq contacts these transcripts through binding of its proximal face to the unstructured U-rich stretches present at the 3′-end of sRNAs. On the other hand, the distal face of the Hfq ring preferentially interacts with ARN motifs in mRNAs. The basic patched rim surface may then interact with UA-rich sites present in both RNAs, coordinating the successful annealing between the sRNA-mRNA pair (Panja et al., 2013; Zhang et al., 2013; Schu et al., 2015). As an example, the iron-responsive class I sRNA RyhB relies on Hfq for successful interaction with its targets (Massé et al., 2003). The less abundant class II sRNAs bind Hfq more tightly, as the interaction is done via the proximal and distal faces of the ring (Panja et al., 2013). Class II sRNAs also use Hfq as a hub for promoting duplex formation to their target mRNAs. This is the case for the MgrR sRNA regulation of the *eptB* and *ygdQ* transcripts (Kwiatkowska et al., 2018). In both sRNA classes, the C-terminal acidic region of Hfq is suggested to help displace unmatched sRNA-mRNA by transiently competing with the core binding surfaces (Woodson et al., 2018). This competition not only allows for a rapid cycling through the pool of cellular RNAs, but also seems to drive substrate RNA specificity in different bacteria (Santiago-Frangos et al., 2017, 2019).

Hfq remodels RNA conformation by disrupting secondary structures, without the need to hydrolyze ATP. This intrinsic RNA chaperone capability is important to unfold structured RNAs, exposing unpaired RNA stretches for basepairing between complementary strands. One of the best characterized Hfq-dependent sRNAs is MicA, which was found to target the *ompA* mRNA (Udekwu et al., 2005). The RNA chaperone activity of Hfq is also important for remodeling MicA structural elements, altering its stability and binding specificities. Hfq binding rearranges MicA fold to allow exposure of the *ompA*-binding site for pairing that leads to translation repression (Andrade et al., 2013; Henderson et al., 2013). Interestingly, target downregulation may require both Hfq and sRNA independently of an sRNA/Hfq complex formation. The two-step regulation of the *dgcM* mRNA was firstly shown to require Hfq to unfold a 5′-end secondary structure that otherwise occludes the binding sites for the OmrA and OmrB sRNAs. Successful binding of OmrA/B to the early coding sequence of *dgcM* results in translation inhibition of the target mRNA (Hoekzema et al., 2019).

### ProQ

ProQ is a recently identified RNA chaperone of the FinO family of RNA-binding proteins commonly found in Proteobacteria (Olejniczak and Storz, 2017). Most of the work on ProQ RNA substrates came from studies performed in *E. coli*, *Salmonella enterica*, and *Legionella pneumophila*, which identified a hundred mRNA transcripts and more than fifty sRNAs as ProQ ligands (Attaiech et al., 2016; Smirnov et al., 2016; Holmqvist et al., 2018; Westermann et al., 2019). ProQ is a monomeric protein with 25 kDa, composed of a α-helical N-terminal domain similar to the RNA-binding domain FinO and a β-sheet C-terminal region partially resembling the eukaryotic Tudor domain. Both regions are connected by a highly flexible and extended linker that is thought to allow the binding and protection of a class of sRNAs that form extended duplexes (Attaiech et al., 2017; Gonzalez et al., 2017; Olejniczak and Storz, 2017). Although both domains contribute to the pairing of complementary RNA molecules, the C-terminal is critical for the RNA strand exchange activity (Chaulk et al., 2011). Unlike Hfq, ProQ binding to RNA is sequence-independent but shows structure preference. ProQ binds double-stranded RNA and prefers highly structured RNAs (Smirnov et al., 2017b; Melamed et al., 2019). The FinO-like domain of ProQ is responsible for this substrate preference (Holmqvist et al., 2018).

Most sRNAs that bind ProQ have unknown functions so far. In contrast to Hfq, the majority of known ProQ-associated sRNAs act *in cis* promoting extensive perfect basepairing with the target mRNA encoded on the opposite strand (Smirnov et al., 2017b). However, ProQ was also found to regulate *trans*-acting sRNAs, assisting the imperfect basepairing with their target mRNAs. Two well characterized examples in *Salmonella* are known: the RaiZ sRNA-*hupA* mRNA and STnc540 sRNA-*mgtB* mRNA (Smirnov et al., 2017b; Westermann et al., 2019). ProQ binds RaiZ through its 3′-terminal stem-loops and promotes interaction of a linear region of this sRNA with the *hupA* mRNA. This three-partner ProQ/RaiZ/*hupA* mRNA complex results in impairment of *hupA* mRNA translation by preventing loading of the 30S ribosome subunit (Smirnov et al., 2017b). STnc540 sRNA also represses the expression of its target mRNA in a ProQ-dependent manner (Westermann et al., 2019). In both examples, ProQ is absolutely required for stability of the sRNAs, affecting their abundance.

Recent work in *E. coli* explores the RNA-RNA interactomes of Hfq and ProQ chaperones using RIL-seq (Melamed et al., 2019). Even though the interactome of ProQ was smaller than the one of Hfq, about a third of the RNA-RNA interactions were common between the two RNA chaperones, with examples like RybB and MalM sRNAs. This suggests complementary or competitive roles for these RBPs. An additional example is found in *Salmonella*, in the regulation mediated by the SraL sRNA. This sRNA binds to the 5′-UTR of the *rho* mRNA, an interaction that can be mediated by ProQ and/or Hfq (Silva et al., 2019). However, the RNAs bound by ProQ generally differ from those bound by Hfq. RIL-seq data revealed that while Hfq-bound RNAs were enriched in both sRNAs and mRNAs, ProQ-bound RNAs were mainly enriched for coding sequences (Melamed et al., 2019). This suggests that the RNA-RNA matchmaking activity of ProQ may not be generalized, unlike observed with Hfq that is primarily involved in sRNA-mediated regulation of mRNA translation. Additional roles for ProQ may include RNA protection from RNase attack or a participation in RNA modification.

### CsrA

The CsrA protein was first discovered in *E. coli* and its function attributed to carbon storage and glycogen production, acting as a translational repressor of the *glgC* mRNA (Romeo et al., 1993; Romeo and Babitzke, 2018). In *Pseudomonas aeruginosa* the homolog protein is termed RsmA (for regulator of secondary metabolism) with paralogs (RsmF/N, RsmE, and RsmI) found in different *Pseudomonas* species (Reimmann et al., 2005; Marden et al., 2013; Morris et al., 2013). Members of the CsrA/RsmA family are conserved among Gammaproteobacteria and have been described as global bacterial regulators (Vakulskas et al., 2015). Here we will focus on the information available on the enterobacterial CsrA.

The *E. coli* CsrA is a ∼7 kDa RNA-binding protein and consists of a homodimer, each subunit with five β-strands, one α-helix and an unstructured C-terminal (Gutiérrez et al., 2005; Duss et al., 2014a). The recognition motif is the AUGGA sequence typically localized in the loop of a stem-loop, as determined by SELEX and confirmed through CLIP-seq (Dubey et al., 2005; Duss et al., 2014a; Holmqvist et al., 2016). The most well characterized activity of CsrA is the binding of mRNA, resulting in repression or activation of translation. Typically, CsrA binding occurs in the RBS sequence or overlaps with the initiation codon, leading to a direct inhibition of translation. CsrA can also regulate transcript stability, either by promoting or blocking the access of the mRNA to ribonucleases (Dubey et al., 2005; Schubert et al., 2007; Yakhnin et al., 2013). CsrA also protects sRNAs from RNase E-mediated degradation, as it was shown for the small RNAs CsrB and CsrC (Weilbacher et al., 2003; Vakulskas et al., 2016). Interestingly, CsrA activity on target mRNAs is mostly regulated by the action of the CsrB and CsrC sRNAs (RsmY and RsmZ in Pseudomonads). These highly structured sRNAs are composed of repetitive sequence elements of the recognition motif GGA (22 per molecule in CsrB and 13 in CsrC) with high affinity for the CsrA binding site (Liu et al., 1997; Weilbacher et al., 2003; Duss et al., 2014b). Consequently, CsrB and CsrC act as “sponges” that sequester CsrA protein and prevent its activity (Romeo et al., 2013). The sRNA McaS is also able to modulate CsrA activity though it contains only two recognition sites (Jørgensen et al., 2013). Transcriptomic studies performed in *E. coli* showed that CsrA affects the abundance of 11 sRNAs, including CsrB and CsrC. Additionally, CLIP-seq analysis followed by *in vitro* studies confirmed CsrA binds other sRNAs with high affinity (Potts et al., 2017). In particular, the interaction of CsrA with the sRNAs GadY, Spot 42, and GcvB was shown to significantly overlap with known basepairing regions for these sRNA–mRNA pairs, suggesting that CsrA binding inhibits formation of these RNA duplexes.

CsrA was recently described to act as an RNA chaperone that indirectly promotes the basepairing between the *trans*-acting SR1 sRNA and its primary target the *ahrC* mRNA, which encodes the transcription activator of the arginine catabolic operons in *Bacillus subtilis* (Müller et al., 2019). *In vitro* binding studies demonstrated that CsrA binds these RNAs with high affinity, in the nanomolar range, even in the presence of an mRNA competitor. Further mutational analysis of the SR1 sRNA and the *ahrC* mRNA confirmed binding of CsrA to both transcripts. CsrA facilitates the binding of the SR1 sRNA downstream the start codon of the *ahrC* mRNA and induces conformational changes in the RBS preventing its translation (Müller et al., 2019). Importantly, Hfq was not found to catalyze this interaction and ProQ is not expressed in *B. subtilis*. Interestingly, this suggests that CsrA may act as an alternative RNA chaperone to Hfq and ProQ in assisting sRNA-mRNA basepairing.

### RNA- and DNA-Binding Multifunctional Proteins as RNA Chaperones

While Hfq, ProQ, and CsrA may be considered the major RNA chaperones interacting with sRNAs, additional RBPs are known to assist RNA folding and bind sRNAs. Two of such examples include the cold shock proteins (CSPs) and the StpA protein. CSPs are a group of small proteins that display the RNA-binding cold shock domain (CSD) (Phadtare and Severinov, 2010) and can passively remodel RNA structures (Woodson et al., 2018). The major cold shock protein of *E. coli* is CspA that binds RNA with low sequence specificity and in a cooperative fashion (Jiang et al., 1997). CspA activity results in the melting of RNA secondary structures, which favors the unfolded state of transcripts enabling their translation (Rennella et al., 2017). In *Staphylococcus aureus*, a RIP-chip assay identified the RNA targets of CspA, which included several sRNAs (Caballero et al., 2018). Accordingly, it is likely that CspA assists sRNA-mediated regulation. Though not all members of the CSP family are induced by cold, they may be relevant for adaptation to other stresses (Yamanaka et al., 1998). For example, CspC and CspE stimulate translation of *rpoS* (encoding the stress sigma factor S) possibly by altering the secondary structure of the *rpoS* mRNA in *E. coli* (Phadtare and Inouye, 2001; Phadtare et al., 2006) and affect virulence in *Salmonella* (Michaux et al., 2017). *E. coli* StpA is another example of an RNA chaperone that remodels RNA structures without hydrolyzing ATP. StpA has RNA annealing and RNA strand displacement activities (Zhang et al., 1995, 1996). It binds weakly to RNA with preference for unstructured molecules, promoting RNA conformational changes by loosening RNA secondary structures (Mayer et al., 2007). Importantly, the RNA chaperone StpA was found to interact with the small RNA MicF. StpA regulates the stability of MicF sRNA and accelerates its base pairing with the target *ompF* mRNA, acting as a major regulator of the OmpF porin expression (Deighan et al., 2000).

## RNases

### RNase E

Homologs of *E. coli* RNase E have been identified in the majority of Proteobacteria classes (Aït-Bara and Carpousis, 2015). This endoribonuclease is composed by a conserved N-terminal catalytic region with an embedded RNA-binding S1 domain, and the unstructured C-terminal non-catalytic region (Bandyra and Luisi, 2018). RNase E cleaves single-stranded RNA, preferably enriched in A/U nucleotides with a stem-loop upstream (Del Campo et al., 2015). Although it prefers substrates with 5′-end monophosphorylated, *in vivo* it is also functional in a 5′-monophosphate-independent pathway (Clarke et al., 2014). Upon Hfq dissociation from the sRNA-mRNA pairs, RNase E can reach and cleave the target mRNA in a linear stretch at the 3′-end of the duplex region (Waters et al., 2017). The sRNAs RyhB and GcvB are typical examples in which sRNA pairing with the coding region promotes mRNA decay via the recruitment of RNase E (Massé et al., 2003; Morita et al., 2005; Lalaouna et al., 2019). Often the base paired sRNA is also degraded with the mRNA. McaS sRNA bound to Hfq interacts with both RNase E and its substrate *csgD* mRNA, leading to the cleavage of both RNAs (Andreassen et al., 2018). RNase E is also critical for the processing of sRNAs from the 3′ UTR of mRNAs, including the release of the CpxQ sRNA from the 3′-end of the *cpxP* mRNA (Chao and Vogel, 2016) and the processing of the precursor RNA to originate the functional ArcZ (Chao et al., 2017). A paralog named RNase G that contains only the catalytic domain of RNase E is also present in *E. coli* and other bacteria (Aït-Bara and Carpousis, 2015). Although this non-essential enzyme shares common activities with RNase E, including rRNA processing and mRNA turnover, no role has been ascribed for RNase G in sRNA processing (Mackie, 2013). Interestingly, RNase E activity on sRNAs can be modulated by other RNA-binding proteins. This is the case of RapZ, an RBP that functions as an adaptor protein in *E. coli*. RapZ binds to the central stem loop of the sRNA GlmZ and RNase E is then recruited for the processing of this sRNA (Göpel et al., 2013), which regulates the *glmS* mRNA encoding the glucosamine-6-phosphate (GlcN6P) synthase. RapZ was recently found to be the receptor for GlcN6P (Khan et al., 2020).

### RNase III

RNase III is a widely distributed endoribonuclease involved in the processing of double-stranded RNAs (dsRNAs). *E. coli* RNase III acts as a 52 kDa homodimer, with the catalytic N-terminal domain connected by a short linker to the C-terminal dsRNA-binding domain (Li and Nicholson, 1996). RNase III can cleave the duplex RNA formed between complementary regions of sRNA and its target mRNA (Lybecker et al., 2014; Altuvia et al., 2018). The target-coupled pathway for RNase III degradation mediated via sRNAs is commonly observed in bacteria. In *Salmonella*, RNase III is responsible for the degradation of the dsRNA formed between MicA and its target *ompA* mRNA upon basepairing (Viegas et al., 2011). In *B. subtilis*, the 3′-end of the antitoxin RatA sRNA forms a large duplex with the *txpA* mRNA that is cleaved by RNase III and prevents the translation of TxpA toxin (Durand et al., 2012). In *Streptococcus pyogenes*, the type II CRISPR-Cas system depends on the maturation of CRISPR RNA by RNase III (Deltcheva et al., 2011).

### PNPase

Polynucleotide phosphorylase (PNPase) is a highly conserved 3′–5′ exoribonuclease that processively degrades RNA (Saramago et al., 2014; Dos Santos et al., 2018). PNPase adopts a homotrimeric organization with a ring-like structure, each monomer having a molecular weight of 78 kDa and holding two RNA-binding domains, KH and S1, on the C-terminal (Shi et al., 2008). In *E. coli*, PNPase is the main enzyme involved in the degradation of sRNAs that are not bound to Hfq, as shown for the regulation of different Hfq-dependent sRNAs, such as MicA, GlmY, RyhB, and SgrS levels (Andrade et al., 2012, 2013). This effect is growth-phase regulated and agrees with previous work in which PNPase was found to degrade sRNAs in the absence of their primary target mRNAs (Andrade and Arraiano, 2008). Additionally, PNPase has an unexpected role in *Listeria monocytogenes*, being responsible for the correct processing of an orphan CRISPR RNA (Sesto et al., 2014).

Although to a lesser extent, additional RNases are involved in the regulation of sRNAs. The degradative enzymes YbeY and RNase R are illustrative examples. YbeY is a highly conserved endoribonuclease commonly associated with rRNA processing (Davies et al., 2010). However, YbeY was shown to bind sRNAs and regulate the levels of sRNAs and mRNAs (Pandey et al., 2011). In *Sinorhizobium meliloti* it was shown that YbeY could cleave sRNA-mRNA pairs (Saramago et al., 2017). Inactivation of YbeY in *E. coli* cells exposed to hydroxyurea resulted in the upregulation of many sRNAs involved in the adaptation to oxidative stress (Pandey et al., 2014). Additionally, *Vibrio cholerae* YbeY was found to regulate the abundance of the sRNAs Qrr1-4 (Vercruysse et al., 2014), which are involved in quorum-sensing and virulence (Tu and Bassler, 2007). RNase R is a unique 3′–5′ exoribonuclease able to degrade highly structured RNAs (Dos Santos et al., 2018). There are few described examples of RNase R involved in the regulation of sRNAs. During cold shock in *E. coli*, RNase R is required for the correct processing of the sRNA SsrA/tmRNA (transfer-messenger RNA), involved in protein quality control and ribosome recycling (Cairrão et al., 2003). RNase R was also described to control sRNA stability of the sRNA SR4 and its target *bsrG* mRNA that together constitute a temperature-dependent type I toxin/antitoxin system in *Bacillus subtilis* (Jahn et al., 2012).

## Conclusion

**FIGURE 2:**
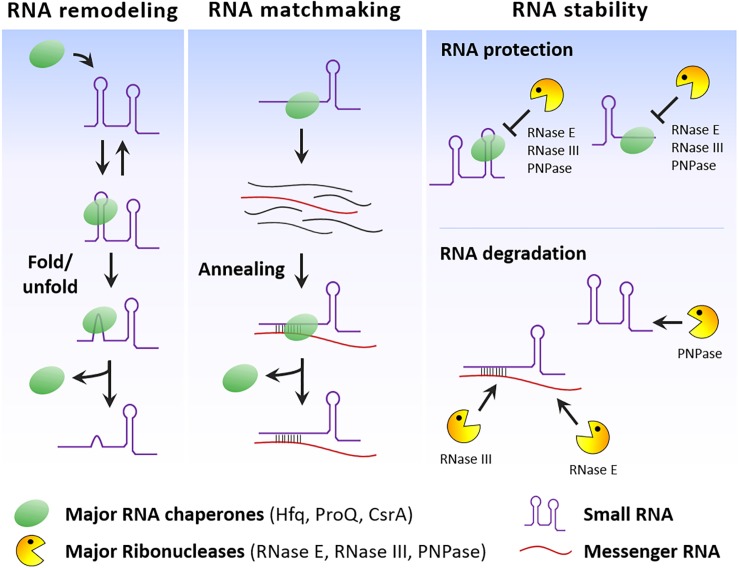
RNA chaperone and RNase activities on sRNAs. Simplified scheme that illustrates the effect of these RNA-binding proteins in different aspects of the sRNA lifetime, namely on the folding of sRNA secondary structures, promotion of sRNA/mRNA basepairing and control of sRNA stability.

RNA chaperones can modify sRNA structure, facilitate the basepairing of sRNAs to their target mRNAs and together with RNases control sRNA stability (Figure 2). However, some RNA chaperones seem to be specific of some species and the activities performed by these regulators may be compensated by other still unidentified RNA-binding proteins. Several RBPs with unorthodox RNA-binding domains have been identified in humans, expanding the number of proteins that can associate with RNA (Castello et al., 2016). Therefore, it is also possible that additional and probably unconventional RBPs interacting with sRNA are going to be discovered in bacteria. The RNA chaperone ProQ offers us a good example of this potential. A new RNA-seq methodology associated to sample fractionation (Grad-seq) contributed to the identification of ProQ as a novel RBP interacting with sRNAs (Smirnov et al., 2016). Application of this and similar methods may contribute to expand the number of sRNA-protein partners and helps to shed light on the many still unknown functions and physiological roles of sRNAs.

## Author Contributions

JA outlined the manuscript. AQ, AS, and RS prepared the figures. CA and JA supervised the work. All authors wrote and participated in preparation of the final manuscript.

## Conflict of Interest

The authors declare that the research was conducted in the absence of any commercial or financial relationships that could be construed as a potential conflict of interest.

## References

[B1] Aït-Bara S. Carpousis A. J. (2015). RNA degradosomes in bacteria and chloroplasts: classification, distribution and evolution of RNase E homologs. *Mol. Microbiol.* 97 1021–1135 10.1111/mmi.13095 26096689

[B2] Altuvia Y. Bar A. Reiss N. Karavani E. Argaman L. Margalit H. (2018). In vivo cleavage rules and target repertoire of RNase III in *Escherichia coli*. *Nucleic Acids Res.* 46 10380–10394. 10.1093/nar/gky684 30113670 PMC6212723

[B3] Andrade J. M. Arraiano C. M. (2008). PNPase is a key player in the regulation of small RNAs that control the expression of outer membrane proteins. *RNA* 14 543–551. 10.1261/rna.683308 18203924 PMC2248267

[B4] Andrade J. M. Dos Santos R. F. Chelysheva I. Ignatova Z. Arraiano C. M. (2018). The RNA-binding protein Hfq is important for ribosome biogenesis and affects translation fidelity. *EMBO J.* 37:e97631. 10.15252/embj.201797631 29669858 PMC5983149

[B5] Andrade J. M. Pobre V. Arraiano C. M. (2013). Small RNA modules confer different stabilities and interact differently with multiple targets. *PLoS One* 8:e52866. 10.1371/journal.pone.0052866 23349691 PMC3551931

[B6] Andrade J. M. Pobre V. Matos A. M. Arraiano C. M. (2012). The crucial role of PNPase in the degradation of small RNAs that are not associated with Hfq. *RNA* 18 844–855. 10.1261/rna.029413.111 22355164 PMC3312570

[B7] Andreassen P. R. Pettersen J. S. Szczerba M. Valentin-Hansen P. Møller-Jensen J. Jørgensen M. G. (2018). sRNA-dependent control of curli biosynthesis in *Escherichia coli*: McaS directs endonucleolytic cleavage of *csg*D mRNA. *Nucleic Acids Res.* 46 6746–6760. 10.1093/nar/gky479 29905843 PMC6061853

[B8] Arraiano C. M. Andrade J. M. Domingues S. Guinote I. B. Malecki M. Matos R. G. (2010). The critical role of RNA processing and degradation in the control of gene expression. *FEMS Microbiol. Rev.* 34 883–923. 10.1111/j.1574-6976.2010.00242.x 20659169

[B9] Attaiech L. Boughammoura A. Brochier-Armanet C. Allatif O. Peillard-Fiorente F. Edwards R. A. (2016). Silencing of natural transformation by an RNA chaperone and a multitarget small RNA. *Proc. Natl. Acad. Sci. U.S.A.* 113 8813–8818. 10.1073/pnas.1601626113 27432973 PMC4978251

[B10] Attaiech L. Glover J. N. M. Charpentier X. (2017). RNA chaperones step out of Hfq’s shadow. *Trends Microbiol.* 25 247–249. 10.1016/j.tim.2017.01.006 28189381

[B11] Bandyra K. J. Luisi B. F. (2018). RNase E and the high-fidelity orchestration of RNA metabolism. *Microbiol. Spectr.* 6:RWR-0008-2017. 10.1128/microbiolspec.RWR-0008-2017 29676248 PMC11633573

[B12] Caballero C. J. Menendez-Gil P. Catalan-Moreno A. Vergara-Irigaray M. García B. Segura V. (2018). The regulon of the RNA chaperone CspA and its auto-regulation in Staphylococcus aureus. *Nucleic Acids Res.* 46 1345–1361. 10.1093/nar/gkx1284 29309682 PMC5815144

[B13] Caillet J. Baron B. Boni I. V. Caillet-Saguy C. Hajnsdorf E. (2019). Identification of protein-protein and ribonucleoprotein complexes containing Hfq. *Sci. Rep.* 9:14054. 10.1038/s41598-019-50562-w 31575967 PMC6773851

[B14] Cairrão F. Cruz A. Mori H. Arraiano C. M. (2003). Cold shock induction of RNase R and its role in the maturation of the quality control mediator SsrA/tmRNA. *Mol. Microbiol.* 50 1349–1360. 10.1046/j.1365-2958.2003.03766.x 14622421

[B15] Castello A. Fischer B. Frese C. K. Horos R. Alleaume A.-M. Foehr S. (2016). Comprehensive identification of RNA-binding domains in human cells. *Mol. Cell* 63 696–710. 10.1016/j.molcel.2016.06.029 27453046 PMC5003815

[B16] Cech G. M. Szalewska-Pałasz A. Kubiak K. Malabirade A. Grange W. Arluison V. (2016). The *Escherichia coli* Hfq protein: an unattended DNA-transactions regulator. *Front. Mol. Biosci.* 3:36. 10.3389/fmolb.2016.00036 27517037 PMC4963395

[B17] Chao Y. Li L. Girodat D. Förstner K. U. Said N. Corcoran C. (2017). *In vivo* cleavage map illuminates the central role of RNase E in coding and non-coding RNA pathways. *Mol. Cell* 65 39–51. 10.1016/j.molcel.2016.11.002 28061332 PMC5222698

[B18] Chao Y. Vogel J. (2016). A 3’ UTR-derived small RNA provides the regulatory noncoding arm of the inner membrane stress response. *Mol. Cell* 61 352–363. 10.1016/j.molcel.2015.12.023 26805574

[B19] Chaulk S. G. Smith Frieday M. N. Arthur D. C. Culham D. E. Edwards R. A. Soo P. (2011). ProQ is an RNA chaperone that controls ProP levels in *Escherichia coli*. *Biochemistry* 50 3095–3106. 10.1021/bi101683a 21381725

[B20] Clarke J. E. Kime L. Romero A. D. McDowall K. J. (2014). Direct entry by RNase E is a major pathway for the degradation and processing of RNA in *Escherichia coli*. *Nucleic Acids Res.* 42 11733–11751. 10.1093/nar/gku808 25237058 PMC4191395

[B21] Davies B. W. Köhrer C. Jacob A. I. Simmons L. A. Zhu J. Aleman L. M. (2010). Role of *Escherichia coli* YbeY, a highly conserved protein, in rRNA processing. *Mol. Microbiol.* 78 506–518. 10.1111/j.1365-2958.2010.07351.x 20807199 PMC2959132

[B22] Deighan P. Free A. Dorman C. J. (2000). A role for the *Escherichia coli* H-NS-like protein StpA in OmpF porin expression through modulation of micF RNA stability. *Mol. Microbiol.* 38 126–139. 10.1046/j.1365-2958.2000.02120.x 11029695

[B23] Del Campo C. Bartholomäus A. Fedyunin I. Ignatova Z. (2015). Secondary structure across the bacterial transcriptome reveals versatile roles in mRNA regulation and function. *PLoS Genet.* 11:e1005613. 10.1371/journal.pgen.1005613 26495981 PMC4619774

[B24] Deltcheva E. Chylinski K. Sharma C. M. Gonzales K. Chao Y. Pirzada Z. A. (2011). CRISPR RNA maturation by trans-encoded small RNA and host factor RNase III. *Nature* 471 602–607. 10.1038/nature09886 21455174 PMC3070239

[B25] Dos Santos R. F. Arraiano C. M. Andrade J. M. (2019). New molecular interactions broaden the functions of the RNA chaperone Hfq. *Curr. Genet.* 65 1313–1319. 10.1007/s00294-019-00990-y 31104083

[B26] Dos Santos R. F. Quendera A. P. Boavida S. Seixas A. F. Arraiano C. M. Andrade J. M. (2018). Major 3’-5’ exoribonucleases in the metabolism of coding and non-coding RNA. *Prog. Mol. Biol. Transl. Sci.* 159 101–155. 10.1016/bs.pmbts.2018.07.005 30340785

[B27] Dubey A. K. Baker C. S. Romeo T. Babitzke P. (2005). RNA sequence and secondary structure participate in high-affinity CsrA-RNA interaction. *RNA* 11 1579–1587. 10.1261/rna.2990205 16131593 PMC1370842

[B28] Durand S. Gilet L. Bessières P. Nicolas P. Condon C. (2012). Three essential ribonucleases-RNase Y, J1, and III-control the abundance of a majority of *Bacillus subtilis* mRNAs. *PLoS Genet.* 8:e1002520. 10.1371/journal.pgen.1002520 22412379 PMC3297567

[B29] Duss O. Michel E. Dit Konté N. D. Schubert M. Allain F. H. T. (2014a). Molecular basis for the wide range of affinity found in Csr/Rsm protein-RNA recognition. *Nucleic Acids Res.* 42 5332–5346. 10.1093/nar/gku141 24561806 PMC4005645

[B30] Duss O. Michel E. Yulikov M. Schubert M. Jeschke G. Allain F. H.-T. (2014b). Structural basis of the non-coding RNA RsmZ acting as a protein sponge. *Nature* 509 588–592. 10.1038/nature13271 24828038

[B31] Fender A. Elf J. Hampel K. Zimmermann B. Wagner E. G. H. (2010). RNAs actively cycle on the Sm-like protein Hfq. *Genes Dev.* 24 2621–2626. 10.1101/gad.591310 21123649 PMC2994036

[B32] Gelsinger D. R. DiRuggiero J. (2018). The non-coding regulatory RNA revolution in archaea. *Genes (Basel)* 9:141. 10.3390/genes9030141 29510582 PMC5867862

[B33] Gonzalez G. M. Hardwick S. W. Maslen S. L. Skehel J. M. Holmqvist E. Vogel J. (2017). Structure of the *Escherichia coli* ProQ RNA-binding protein. *RNA* 23 696–711. 10.1261/rna.060343.116 28193673 PMC5393179

[B34] Göpel Y. Papenfort K. Reichenbach B. Vogel J. Görke B. (2013). Targeted decay of a regulatory small RNA by an adaptor protein for RNase E and counteraction by an anti-adaptor RNA. *Genes Dev.* 27 552–564. 10.1101/gad.210112.112 23475961 PMC3605468

[B35] Gutiérrez P. Li Y. Osborne M. J. Pomerantseva E. Liu Q. Gehring K. (2005). Solution structure of the carbon storage regulator protein CsrA from *Escherichia coli*. *J. Bacteriol.* 187 3496–3501. 10.1128/JB.187.10.3496-3501.2005 15866937 PMC1112004

[B36] Henderson C. A. Vincent H. A. Stone C. M. Phillips J. O. Cary P. D. Gowers D. M. (2013). Characterization of MicA interactions suggests a potential novel means of gene regulation by small non-coding RNAs. *Nucleic Acids Res.* 41 3386–3397. 10.1093/nar/gkt008 23361466 PMC3597676

[B37] Hoekzema M. Romilly C. Holmqvist E. Wagner E. G. H. (2019). Hfq−dependent mRNA unfolding promotes sRNA −based inhibition of translation. *EMBO J.* 38:e101199. 10.15252/embj.2018101199 30833291 PMC6443205

[B38] Holmqvist E. Li L. Bischler T. Barquist L. Vogel J. (2018). Global maps of ProQ binding In Vivo reveal target recognition via RNA structure and stability control at mRNA 3’ ends. *Mol. Cell* 70 971–982.e6. 10.1016/j.molcel.2018.04.017 29804828

[B39] Holmqvist E. Vogel J. (2018). RNA-binding proteins in bacteria. *Nat. Rev. Microbiol.* 16 601–615. 10.1038/s41579-018-0049-5 29995832

[B40] Holmqvist E. Wright P. R. Li L. Bischler T. Barquist L. Reinhardt R. (2016). Global RNA recognition patterns of post-transcriptional regulators Hfq and CsrA revealed by UV crosslinking *in vivo*. *EMBO J.* 35 991–1011. 10.15252/embj.201593360 27044921 PMC5207318

[B41] Hwang W. Arluison V. Hohng S. (2011). Dynamic competition of DsrA and rpoS fragments for the proximal binding site of Hfq as a means for efficient annealing. *Nucleic Acids Res.* 39 5131–5139. 10.1093/nar/gkr075 21357187 PMC3130260

[B42] Jahn N. Preis H. Wiedemann C. Brantl S. (2012). BsrG/SR4 from *Bacillus subtilis* - the first temperature-dependent type I toxin-antitoxin system. *Mol. Microbiol.* 83 579–598. 10.1111/j.1365-2958.2011.07952.x 22229825

[B43] Jiang K. Zhang C. Guttula D. Liu F. Van Kan J. A. Lavelle C. (2015). Effects of Hfq on the conformation and compaction of DNA. *Nucleic Acids Res.* 43 4332–4341. 10.1093/nar/gkv268 25824948 PMC4417175

[B44] Jiang W. Hou Y. Inouye M. (1997). CspA, the major cold-shock protein of *Escherichia coli*, is an RNA chaperone. *J. Biol. Chem.* 272 196–202. 10.1074/jbc.272.1.196 8995247

[B45] Jørgensen M. G. Thomason M. K. Havelund J. Valentin-Hansen P. Storz G. (2013). Dual function of the McaS small RNA in controlling biofilm formation. *Genes Dev.* 27 1132–1145. 10.1101/gad.214734.113 23666921 PMC3672647

[B46] Khan M. A. Durica-Mitic S. Göpel Y. Heermann R. Görke B. (2020). Small RNA-binding protein RapZ mediates cell envelope precursor sensing and signaling in *Escherichia coli*. *EMBO J.* 39:e103848. 10.15252/embj.2019103848 32065419 PMC7073468

[B47] Kwiatkowska J. Wroblewska Z. Johnson K. A. Olejniczak M. (2018). The binding of Class II sRNA MgrR to two different sites on matchmaker protein Hfq enables efficient competition for Hfq and annealing to regulated mRNAs. *RNA* 24 1761–1784. 10.1261/rna.067777.118 30217864 PMC6239178

[B48] Lalaouna D. Eyraud A. Devinck A. Prévost K. Massé E. (2019). GcvB small RNA uses two distinct seed regions to regulate an extensive targetome. *Mol. Microbiol.* 111 473–486. 10.1111/mmi.14168 30447071

[B49] Le Derout J. Folichon M. Briani F. Dehò G. Régnier P. Hajnsdorf E. (2003). Hfq affects the length and the frequency of short oligo(A) tails at the 3’ end of *Escherichia coli rps*O mRNAs. *Nucleic Acids Res.* 31 4017–4023. 10.1093/nar/gkg456 12853618 PMC165971

[B50] Lee T. Feig A. L. (2008). The RNA binding protein Hfq interacts specifically with tRNAs. *RNA* 14 514–523. 10.1261/rna.531408 18230766 PMC2248270

[B51] Li H. Nicholson A. W. (1996). Defining the enzyme binding domain of a ribonuclease III processing signal. Ethylation interference and hydroxyl radical footprinting using catalytically inactive RNase III mutants. *EMBO J.* 15 1421–1433. 10.1002/j.1460-2075.1996.tb00484.x 8635475 PMC450047

[B52] Link T. M. Valentin-Hansen P. Brennan R. G. (2009). Structure of *Escherichia coli* Hfq bound to polyriboadenylate RNA. *Proc. Natl. Acad. Sci. U.S.A.* 106 19292–19297. 10.1073/pnas.0908744106 19889981 PMC2773200

[B53] Liu M. Y. Gui G. Wei B. Preston J. F. Oakford L. Yüksel U. (1997). The RNA molecule CsrB binds to the global regulatory protein CsrA and antagonizes its activity in *Escherichia coli*. *J. Biol. Chem.* 272 17502–17510. 10.1074/jbc.272.28.17502 9211896

[B54] Lybecker M. Zimmermann B. Bilusic I. Tukhtubaeva N. Schroeder R. (2014). The double-stranded transcriptome of *Escherichia coli*. *Proc. Natl. Acad. Sci. U.S.A.* 111 3134–3139. 10.1073/pnas.1315974111 24453212 PMC3939876

[B55] Mackie G. A. (2013). RNase E: at the interface of bacterial RNA processing and decay. *Nat. Rev. Microbiol.* 11 45–57. 10.1038/nrmicro2930 23241849

[B56] Malabirade A. Jiang K. Kubiak K. Diaz-Mendoza A. Liu F. van Kan J. A. (2017). Compaction and condensation of DNA mediated by the C-terminal domain of Hfq. *Nucleic Acids Res.* 45 7299–7308. 10.1093/nar/gkx431 28521053 PMC5499573

[B57] Marden J. N. Diaz M. R. Walton W. G. Gode C. J. Betts L. Urbanowski M. L. (2013). An unusual CsrA family member operates in series with RsmA to amplify posttranscriptional responses in *Pseudomonas aeruginosa*. *Proc. Natl. Acad. Sci. U.S.A.* 110 15055–15060. 10.1073/pnas.1307217110 23980177 PMC3773774

[B58] Massé E. Escorcia F. E. Gottesman S. (2003). Coupled degradation of a small regulatory RNA and its mRNA targets in *Escherichia coli*. *Genes Dev.* 17 2374–2383. 10.1101/gad.1127103 12975324 PMC218075

[B59] Mayer O. Rajkowitsch L. Lorenz C. Konrat R. Schroeder R. (2007). RNA chaperone activity and RNA-binding properties of the *E. coli* protein StpA. *Nucleic Acids Res.* 35 1257–1269. 10.1093/nar/gkl1143 17267410 PMC1851640

[B60] Melamed S. Adams P. P. Zhang A. Zhang H. Storz G. (2019). RNA-RNA Interactomes of ProQ and Hfq reveal overlapping and competing roles. *Mol. Cell* 77 411–425.e7. 10.1016/j.molcel.2019.10.022 31761494 PMC6980735

[B61] Melamed S. Peer A. Faigenbaum-Romm R. Gatt Y. E. Reiss N. Bar A. (2016). Global mapping of small RNA-target interactions in bacteria. *Mol. Cell* 63 884–897. 10.1016/j.molcel.2016.07.026 27588604 PMC5145812

[B62] Michaux C. Holmqvist E. Vasicek E. Sharan M. Barquist L. Westermann A. J. (2017). RNA target profiles direct the discovery of virulence functions for the cold-shock proteins CspC and CspE. *Proc. Natl. Acad. Sci. U.S.A.* 114 6824–6829. 10.1073/pnas.1620772114 28611217 PMC5495234

[B63] Mihailovic M. K. Vazquez-Anderson J. Li Y. Fry V. Vimalathas P. Herrera D. (2018). High-throughput *in vivo* mapping of RNA accessible interfaces to identify functional sRNA binding sites. *Nat. Commun.* 9:4084. 10.1038/s41467-018-06207-z 30287822 PMC6172242

[B64] Mohanty B. K. Maples V. F. Kushner S. R. (2004). The Sm-like protein Hfq regulates polyadenylation dependent mRNA decay in *Escherichia coli*. *Mol. Microbiol.* 54 905–920. 10.1111/j.1365-2958.2004.04337.x 15522076

[B65] Møller T. Franch T. Højrup P. Keene D. R. Bächinger H. P. Brennan R. G. (2002). Hfq: a bacterial Sm-like protein that mediates RNA-RNA interaction. *Mol. Cell* 9 23–30. 10.1016/s1097-2765(01)00436-1 11804583

[B66] Morita T. Maki K. Aiba H. (2005). RNase E-based ribonucleoprotein complexes: mechanical basis of mRNA destabilization mediated by bacterial noncoding RNAs. *Genes Dev.* 19 2176–2186. 10.1101/gad.1330405 16166379 PMC1221888

[B67] Morris E. R. Hall G. Li C. Heeb S. Kulkarni R. V. Lovelock L. (2013). Structural rearrangement in an RsmA/CsrA Ortholog of *Pseudomonas aeruginosa* creates a dimeric RNA-binding protein, RsmN. *Structure* 21 1659–1671. 10.1016/j.str.2013.07.007 23954502 PMC3791407

[B68] Müller P. Gimpel M. Wildenhain T. Brantl S. (2019). A new role for CsrA: promotion of complex formation between an sRNA and its mRNA target in *Bacillus subtilis*. *RNA Biol.* 16 972–987. 10.1080/15476286.2019.1605811 31043113 PMC6546359

[B69] Olejniczak M. Storz G. (2017). ProQ/FinO-domain proteins: another ubiquitous family of RNA matchmakers? *Mol. Microbiol.* 104 905–915. 10.1111/mmi.13679 28370625 PMC5578414

[B70] Pandey S. P. Minesinger B. K. Kumar J. Walker G. C. (2011). A highly conserved protein of unknown function in *Sinorhizobium meliloti* affects sRNA regulation similar to Hfq. *Nucleic Acids Res.* 39 4691–4708. 10.1093/nar/gkr060 21325267 PMC3113577

[B71] Pandey S. P. Winkler J. A. Li H. Camacho D. M. Collins J. J. Walker G. C. (2014). Central role for RNase YbeY in Hfq-dependent and Hfq-independent small-RNA regulation in bacteria. *BMC Genomics* 15:121. 10.1186/1471-2164-15-121 24511998 PMC3933206

[B72] Panja S. Schu D. J. Woodson S. A. (2013). Conserved arginines on the rim of Hfq catalyze base pair formation and exchange. *Nucleic Acids Res.* 41 7536–7546. 10.1093/nar/gkt521 23771143 PMC3753642

[B73] Phadtare S. Inouye M. (2001). Role of CspC and CspE in regulation of expression of RpoS and UspA, the stress response proteins in *Escherichia coli*. *J. Bacteriol.* 183 1205–1214. 10.1128/JB.183.4.1205-1214.2001 11157932 PMC94993

[B74] Phadtare S. Severinov K. (2010). RNA remodeling and gene regulation by cold shock proteins. *RNA Biol.* 7 788–795. 10.4161/rna.7.6.13482 21045540 PMC3073336

[B75] Phadtare S. Tadigotla V. Shin W. H. Sengupta A. Severinov K. (2006). Analysis of *Escherichia coli* global gene expression profiles in response to overexpression and deletion of CspC and CspE. *J. Bacteriol.* 188 2521–2527. 10.1128/JB.188.7.2521-2527.2006 16547039 PMC1428408

[B76] Potts A. H. Vakulskas C. A. Pannuri A. Yakhnin H. Babitzke P. Romeo T. (2017). Global role of the bacterial post-transcriptional regulator CsrA revealed by integrated transcriptomics. *Nat. Commun.* 8:1596. 10.1038/s41467-017-01613-1 29150605 PMC5694010

[B77] Rajkowitsch L. Schroeder R. (2007). Dissecting RNA chaperone activity. *RNA* 13 2053–2060. 10.1261/rna.671807 17901153 PMC2080586

[B78] Reimmann C. Valverde C. Kay E. Haas D. (2005). Posttranscriptional repression of GacS/GacA-controlled genes by the RNA-binding protein RsmE acting together with RsmA in the biocontrol strain *Pseudomonas fluorescens* CHA0. *J. Bacteriol.* 187 276–285. 10.1128/JB.187.1.276-285.2005 15601712 PMC538806

[B79] Rennella E. Sára T. Juen M. Wunderlich C. Imbert L. Solyom Z. (2017). RNA binding and chaperone activity of the *E. coli* cold-shock protein CspA. *Nucleic Acids Res.* 45 4255–4268. 10.1093/nar/gkx044 28126922 PMC5397153

[B80] Romeo T. Babitzke P. (2018). Global Regulation by CsrA and Its RNA Antagonists. *Microbiol. Spectr.* 6:RWR–0009–2017. 10.1128/microbiolspec.rwr-0009-2017 29573256 PMC5868435

[B81] Romeo T. Gong M. Liu M. Y. Brun-Zinkernagel A. M. (1993). Identification and molecular characterization of *csrA*, a pleiotropic gene from *Escherichia coli* that affects glycogen biosynthesis, gluconeogenesis, cell size, and surface properties. *J. Bacteriol.* 175 4744–4755. 10.1128/jb.175.15.4744-4755.1993 8393005 PMC204926

[B82] Romeo T. Vakulskas C. A. Babitzke P. (2013). Post-transcriptional regulation on a global scale: form and function of Csr/Rsm systems. *Environ. Microbiol.* 15 313–324. 10.1111/j.1462-2920.2012.02794.x 22672726 PMC3443267

[B83] Santiago-Frangos A. Fröhlich K. S. Jeliazkov J. R. Małecka E. M. Marino G. Gray J. J. (2019). Caulobacter crescentus Hfq structure reveals a conserved mechanism of RNA annealing regulation. *Proc. Natl. Acad. Sci. U.S.A.* 166 10978–10987. 10.1073/pnas.1814428116 31076551 PMC6561178

[B84] Santiago-Frangos A. Jeliazkov J. R. Gray J. J. Woodson S. A. (2017). Acidic C-terminal domains autoregulate the RNA chaperone Hfq. *eLife* 6:e27049. 10.7554/eLife.27049 28826489 PMC5606850

[B85] Santiago-Frangos A. Woodson S. A. (2018). Hfq chaperone brings speed dating to bacterial sRNA. *Wiley Interdiscip. Rev. RNA* 9:e1475. 10.1002/wrna.1475 29633565 PMC6002925

[B86] Saramago M. Bárria C. Dos Santos R. F. Silva I. J. Pobre V. Domingues S. (2014). The role of RNases in the regulation of small RNAs. *Curr. Opin. Microbiol.* 18 105–115. 10.1016/j.mib.2014.02.009 24704578

[B87] Saramago M. Peregrina A. Robledo M. Matos R. G. Hilker R. Serrania J. (2017). *Sinorhizobium meliloti* YbeY is an endoribonuclease with unprecedented catalytic features, acting as silencing enzyme in riboregulation. *Nucleic Acids Res.* 45 1371–1391. 10.1093/nar/gkw1234 28180335 PMC5388416

[B88] Sauer E. Schmidt S. Weichenrieder O. (2012). Small RNA binding to the lateral surface of Hfq hexamers and structural rearrangements upon mRNA target recognition. *Proc. Natl. Acad. Sci. U.S.A.* 109 9396–9401. 10.1073/pnas.1202521109 22645344 PMC3386104

[B89] Sauer E. Weichenrieder O. (2011). Structural basis for RNA 3’-end recognition by Hfq. *Proc. Natl. Acad. Sci. U.S.A.* 108 13065–13070. 10.1073/pnas.1103420108 21737752 PMC3156190

[B90] Schu D. J. Zhang A. Gottesman S. Storz G. (2015). Alternative Hfq-sRNA interaction modes dictate alternative mRNA recognition. *EMBO J.* 34 2557–2573. 10.15252/embj.201591569 26373314 PMC4609186

[B91] Schubert M. Lapouge K. Duss O. Oberstrass F. C. Jelesarov I. Haas D. (2007). Molecular basis of messenger RNA recognition by the specific bacterial repressing clamp RsmA/CsrA. *Nat. Struct. Mol. Biol.* 14 807–813. 10.1038/nsmb1285 17704818

[B92] Sesto N. Touchon M. Andrade J. M. Kondo J. Rocha E. P. C. Arraiano C. M. (2014). A PNPase dependent CRISPR System in Listeria. *PLoS Genet.* 10:e1004065. 10.1371/journal.pgen.1004065 24415952 PMC3886909

[B93] Shi Z. Yang W.-Z. Lin-Chao S. Chak K.-F. Yuan H. S. (2008). Crystal structure of *Escherichia coli* PNPase: central channel residues are involved in processive RNA degradation. *RNA* 14 2361–2371. 10.1261/rna.1244308 18812438 PMC2578853

[B94] Shimoni Y. Friedlander G. Hetzroni G. Niv G. Altuvia S. Biham O. (2007). Regulation of gene expression by small non-coding RNAs: a quantitative view. *Mol. Syst. Biol.* 3:138. 10.1038/msb4100181 17893699 PMC2013925

[B95] Silva I. J. Barahona S. Eyraud A. Lalaouna D. Figueroa-Bossi N. Massé E. (2019). SraL sRNA interaction regulates the terminator by preventing premature transcription termination of *rho* mRNA. *Proc. Natl. Acad. Sci. U.S.A.* 116 3042–3051. 10.1073/pnas.1811589116 30718400 PMC6386699

[B96] Smirnov A. Förstner K. U. Holmqvist E. Otto A. Günster R. Becher D. (2016). Grad-seq guides the discovery of ProQ as a major small RNA-binding protein. *Proc. Natl. Acad. Sci. U.S.A.* 113 11591–11596. 10.1073/pnas.1609981113 27671629 PMC5068311

[B97] Smirnov A. Schneider C. Hör J. Vogel J. (2017a). Discovery of new RNA classes and global RNA-binding proteins. *Curr. Opin. Microbiol.* 39 152–160. 10.1016/j.mib.2017.11.016 29179042

[B98] Smirnov A. Wang C. Drewry L. L. Vogel J. (2017b). Molecular mechanism of mRNA repression *in trans* by a ProQ-dependent small RNA. *EMBO J.* 36 1029–1045. 10.15252/embj.201696127 28336682 PMC5391140

[B99] Storz G. Vogel J. Wassarman K. M. (2011). Regulation by small RNAs in bacteria: expanding frontiers. *Mol. Cell* 43 880–891. 10.1016/j.molcel.2011.08.022 21925377 PMC3176440

[B100] Szklarczyk D. Morris J. H. Cook H. Kuhn M. Wyder S. Simonovic M. (2017). The STRING database in 2017: quality-controlled protein-protein association networks, made broadly accessible. *Nucleic Acids Res.* 45 D362–D368. 10.1093/nar/gkw937 27924014 PMC5210637

[B101] Tu K. C. Bassler B. L. (2007). Multiple small RNAs act additively to integrate sensory information and control quorum sensing in *Vibrio harveyi*. *Genes Dev.* 21 221–233. 10.1101/gad.1502407 17234887 PMC1770904

[B102] Udekwu K. I. Darfeuille F. Vogel J. Reimegård J. Holmqvist E. Wagner E. G. H. (2005). Hfq-dependent regulation of OmpA synthesis is mediated by an antisense RNA. *Genes Dev.* 19 2355–2366. 10.1101/gad.354405 16204185 PMC1240044

[B103] Vakulskas C. A. Leng Y. Abe H. Amaki T. Okayama A. Babitzke P. (2016). Antagonistic control of the turnover pathway for the global regulatory sRNA CsrB by the CsrA and CsrD proteins. *Nucleic Acids Res.* 44 7896–7910. 10.1093/nar/gkw484 27235416 PMC5027483

[B104] Vakulskas C. A. Potts A. H. Babitzke P. Ahmer B. M. M. Romeo T. (2015). Regulation of Bacterial Virulence by Csr (Rsm) Systems. *Microbiol. Mol. Biol. Rev.* 79 193–224. 10.1128/mmbr.00052-14 25833324 PMC4394879

[B105] Vercruysse M. Köhrer C. Davies B. W. Arnold M. F. F. Mekalanos J. J. RajBhandary U. L. (2014). The highly conserved bacterial RNase YbeY is essential in *Vibrio cholerae*, playing a critical role in virulence, stress regulation, and RNA processing. *PLoS Pathog.* 10:e1004175. 10.1371/journal.ppat.1004175 24901994 PMC4047096

[B106] Viegas S. C. Silva I. J. Saramago M. Domingues S. Arraiano C. M. (2011). Regulation of the small regulatory RNA MicA by ribonuclease III: a target-dependent pathway. *Nucleic Acids Res.* 39 2918–2930. 10.1093/nar/gkq1239 21138960 PMC3074148

[B107] Vogel J. Luisi B. F. (2011). Hfq and its constellation of RNA. *Nat. Rev. Microbiol.* 9 578–589. 10.1038/nrmicro2615 21760622 PMC4615618

[B108] Waters S. A. McAteer S. P. Kudla G. Pang I. Deshpande N. P. Amos T. G. (2017). Small RNA interactome of pathogenic *E. coli* revealed through crosslinking of RNase E. *EMBO J.* 36 374–387. 10.15252/embj.201694639 27836995 PMC5286369

[B109] Weilbacher T. Suzuki K. Dubey A. K. Wang X. Gudapaty S. Morozov I. (2003). A novel sRNA component of the carbon storage regulatory system of *Escherichia coli*. *Mol. Microbiol.* 48 657–670. 10.1046/j.1365-2958.2003.03459.x 12694612

[B110] Westermann A. J. Venturini E. Sellin M. E. Förstner K. U. Hardt W.-D. Vogel J. (2019). The Major RNA-Binding Protein ProQ Impacts Virulence Gene Expression in *Salmonella enterica* Serovar Typhimurium. *mBio* 10:e02504-18. 10.1128/mBio.02504-18 30602583 PMC6315103

[B111] Woodson S. A. Panja S. Santiago-Frangos A. (2018). Proteins That Chaperone RNA Regulation. *Microbiol. Spectr.* 6 385–397. 10.1128/microbiolspec.RWR-0026-2018 30051798 PMC6086601

[B112] Yakhnin A. V. Baker C. S. Vakulskas C. A. Yakhnin H. Berezin I. Romeo T. (2013). CsrA activates *flhDC* expression by protecting flhDC mRNA from RNase E-mediated cleavage. *Mol. Microbiol.* 87 851–866. 10.1111/mmi.12136 23305111 PMC3567230

[B113] Yamanaka K. Fang L. Inouye M. (1998). The CspA family in *Escherichia coli*: multiple gene duplication for stress adaptation. *Mol. Microbiol.* 27 247–255. 10.1046/j.1365-2958.1998.00683.x 9484881

[B114] Zhang A. Derbyshire V. Galloway Salvo J. L. Belfort M. (1995). *Escherichia coli* protein StpA stimulates self-splicing by promoting RNA assembly in vitro. *RNA* 1 783–793. 7493324 PMC1369319

[B115] Zhang A. Rimsky S. Reaban M. E. Buc H. Belfort M. (1996). *Escherichia coli* protein analogs StpA and H-NS: regulatory loops, similar and disparate effects on nucleic acid dynamics. *EMBO J.* 15 1340–1349. 10.1002/j.1460-2075.1996.tb00476.x 8635467 PMC450038

[B116] Zhang A. Schu D. J. Tjaden B. C. Storz G. Gottesman S. (2013). Mutations in interaction surfaces differentially impact *E. coli* Hfq association with small RNAs and their mRNA targets. *J. Mol. Biol.* 425 3678–3697. 10.1016/j.jmb.2013.01.006 23318956 PMC3640674

